# Recent Advances in Self-Powered Wearable Flexible Sensors for Human Gaits Analysis

**DOI:** 10.3390/nano14141173

**Published:** 2024-07-10

**Authors:** Xiaohe Hu, Zhiqiang Ma, Fuqun Zhao, Sheng Guo

**Affiliations:** 1 School of Mechanical, Electronic and Control Engineering, Beijing Jiaotong University, Beijing 100044, China; xhhu@bjtu.edu.cn (X.H.); fqzhao@buaa.edu.cn (F.Z.); 2 Department of Biomedical Engineering, City University of Hong Kong, 83 Tat Chee Avenue, Kowloon, Hong Kong 999077, China

**Keywords:** exoskeleton, wearable flexible sensors, robotics, motion detection

## Abstract

The rapid progress of flexible electronics has met the growing need for detecting human movement information in exoskeleton auxiliary equipment. This study provides a review of recent advancements in the design and fabrication of flexible electronics used for human motion detection. Firstly, a comprehensive introduction is provided on various self-powered wearable flexible sensors employed in detecting human movement information. Subsequently, the algorithms utilized to provide feedback on human movement are presented, followed by a thorough discussion of their methods and effectiveness. Finally, the review concludes with perspectives on the current challenges and opportunities in implementing self-powered wearable flexible sensors in exoskeleton technology.

## 1. Introduction

In the past few decades, there has been a notable surge in interest and attention towards the concept of lower limb exoskeletons [1,2,3,4,5]. These extraordinary devices, intended to be worn externally, act as extensions or enhancements of the human body, catering to diverse application scenarios encompassing medical science, industry, and military applications [6,7,8]. Broadly speaking, the role of exoskeletons can be summarized as offering assistance for walking and auxiliary support [9,10].

The integration of human gait detection is essential for the effective utilization of exoskeletons in assisting human walking. Human gait, a complex biomechanical process, entails the coordinated movement of multiple body segments [11,12,13]. Each individual exhibits a unique gait pattern influenced by various factors such as age, gender, body mass, and musculoskeletal health [14,15]. A comprehensive understanding of human gait features holds significant importance in assessing and monitoring movement information for exoskeletons, as well as in the clinical diagnosis and treatment of gait-related disorders [16,17,18,19].

Sensors assume a fundamental role in the perception and recognition of human gait [20,21,22]. They enable the indispensable acquisition of data that inform the movements of the exoskeleton, as well as its interactions with the wearer and the environment. At the heart of every exoskeleton system is a network of sensors that continuously monitor a wide range of parameters, encompassing joint movements [23,24,25], force [26,27], and inertia [28,29,30]. The strategic placement of each sensor is crucial in capturing the most relevant information for a specific function or movement.

Traditionally, exoskeletons have heavily relied on rigid sensors, which can be bulky, cumbersome, and uncomfortable to wear for extended periods [31,32,33,34,35]. However, in recent years, remarkable advancements have been made in the development of wearable flexible sensors based on various working principles, including piezoresistive [36,37], capacitive [38,39], piezoelectric [40,41,42], and triboelectric [43,44,45,46,47,48,49,50,51]. Among these, piezoelectric and triboelectric sensors have emerged as highly promising detection methods due to their ability to operate without the need for an external power source [52,53,54,55,56]. This unique characteristic renders them an ideal choice for continuous and long-term monitoring of human motion, thereby offering significant potential for exoskeleton applications [57,58,59,60].

The application of gait assessment algorithms on lower limb exoskeletons has gained significant importance with the advancement of artificial intelligence and machine learning technologies [61,62,63,64]. By leveraging sensors and advanced algorithm techniques, it becomes possible to monitor the user’s gait characteristics, including stride length, cadence, stance time, and more, in real time. These data facilitate exoskeleton systems in adjusting support levels and gait patterns according to individual needs, thereby enhancing walking efficiency and comfort for the user. Moreover, when combined with precise gait analysis, medical professionals can evaluate rehabilitation progress, develop personalized rehabilitation plans, and optimize the outcome of rehabilitation [65,66,67]. The integration of gait assessment algorithms in the realm of lower limb exoskeletons opens up new possibilities for rehabilitation and assisted walking, perpetually propelling innovation and advancement in this field.

**Figure 1:**
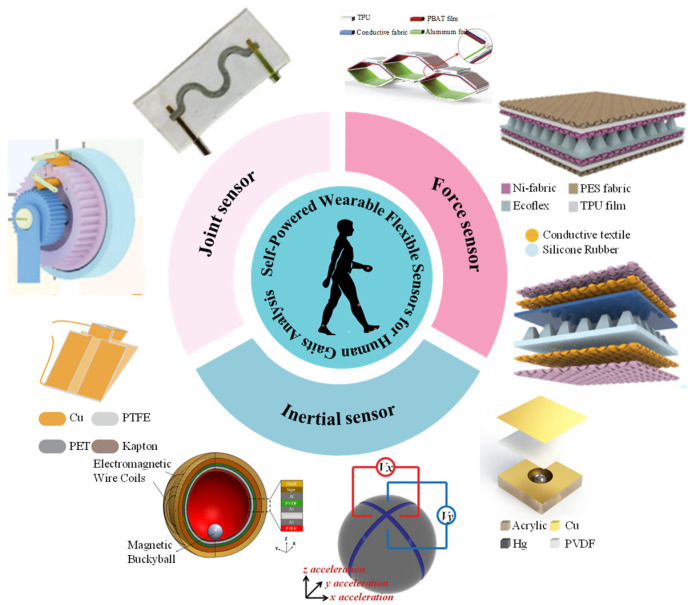
Schematic diagram of the self-powered wearable flexible sensors for human gaits analysis. Reproduced with permission from Ref. [68]. Copyright 2021, Science. Reproduced with permission from Ref. [69]. Copyright 2024, Wiley. Reproduced with permission from Ref. [70]. Copyright 2023, Wiley. Reproduced with permission from Ref. [71]. Copyright 2021, Wiley. Reproduced with permission from Ref. [72]. Copyright 2022, Wiley. Reproduced with permission from Ref. [73]. Copyright 2020, Springer Nature. Reproduced with permission. from Ref. [74]. Copyright 2017, American Chemistry Society. Reproduced with permission from Ref. [75]. Copyright 2019, Elsevier. Reproduced with permission from Ref. [76]. Copyright 2017, Wiley.

The structure of this review paper is tailored according to Figure 1, as outlined below: Firstly, we conduct an analysis and summary of the recent advancements in self-powered wearable flexible sensors, specifically focusing on piezoelectric nanogenerators (PENGs) and triboelectric nanogenerators (TENGs), with applications in joint motion, force, and inertia detection. Subsequently, we delve into the discussion of gait assessment algorithms, encompassing various types of algorithms and their diverse applications. Finally, we provide a comprehensive overview of the challenges and perspectives pertaining to the utilization of sensors in exoskeleton technology.

## 2. Self-Powered Wearable Flexible Sensors for Gaits Analysis

### 2.1. Self-Powered Sensing Mechanisms and Nanomaterials Design 

**Figure 2:**
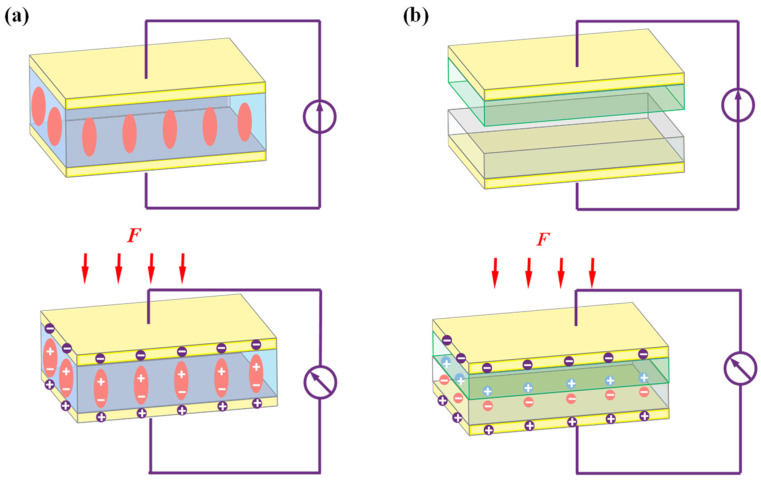
Schematic illustrations of transduction principles of self-powered wearable flexible sensors for human gaits analysis: (**a**) piezoelectricity and (**b**) triboelectricity.

Scientists have made significant advancements in the development of self-powered wearable flexible sensors for healthcare, gait detection, and analysis. These sensors leverage diverse sensing mechanisms, including piezoelectricity [40], triboelectricity [77], thermoelectricity [78], pyroelectricity [79], solar cells [44], and electromagnetic sensing [80], among others. In this comprehensive review, our focus primarily centers on summarizing and discussing self-powered wearable flexible sensors specifically based on piezoelectricity and triboelectricity for the detection and analysis of gait patterns. As illustrated in Figure 2a,b, piezoelectricity and triboelectricity working principles are widely employed to develop the self-powered wearable flexible sensors for detecting human motion. Piezoelectric sensors leverage the piezoelectric effect exhibited by non-centrosymmetric dipole structures in specific materials, enabling the conversion of mechanical stimuli from joints into electrical signals [81]. Conversely, TENGs generate electric charge through the contact and separation of materials possessing distinct triboelectric properties [82,83]. These sensors facilitate noninvasive and continuous monitoring of joint movements, thereby providing valuable insights into an individual’s motor activities. Their remarkable sensitivity and precision render them highly suitable for applications in both clinical and research settings, where in-depth analysis of locomotor patterns is imperative. Moreover, the integration of piezoelectric and triboelectric sensors into exoskeletons offers notable advantages in terms of portability and real-time data acquisition, thereby facilitating advanced studies in biomechanics, rehabilitation, and human–computer interaction.

**Figure 3:**
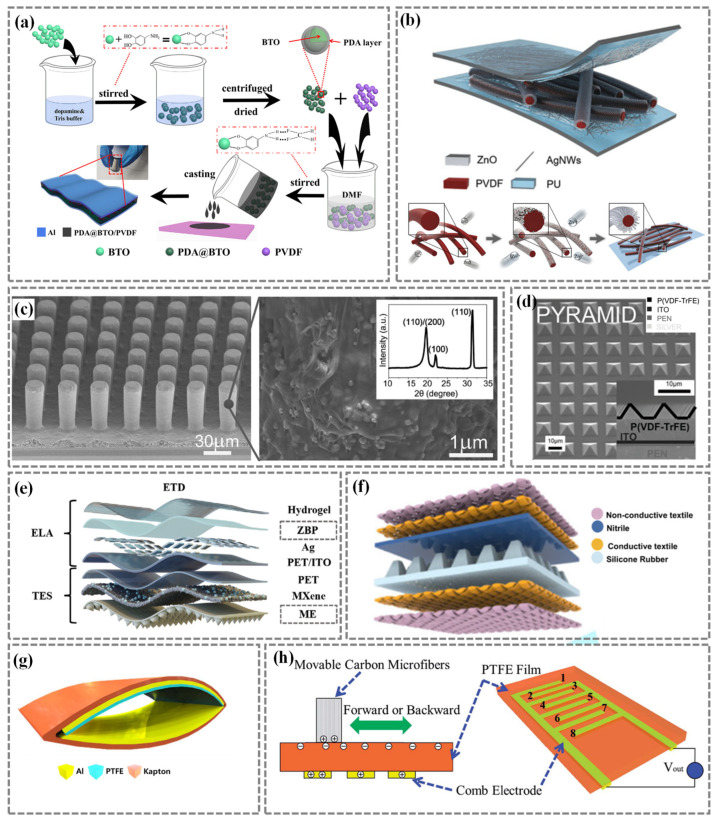
Enhancement methods for piezoelectric nanogenerators (PENGs) and triboelectric nanogenerators (TENGs). (**a**) Fabrication process of the polydopamine (PDA)-modified barium titanate (BTO)/poly(vinylidene fluoride) (PVDF) composite film. Reproduced with permission from Ref. [87]. Copyright 2020, Elsevier. (**b**) Three-dimensional hierarchically interlocked PVDF/ZnO fibers-based physiological monitoring electronics. Reproduced with permission from Ref. [93]. Copyright 2020, Elsevier. (**c**) Scanning electron microscopy (SEM) image of polyvinylidene fluoride-trifluoroethylen [P(VDF-TrFE)]/BTO nanocomposite micropillar array. Reproduced with permission from Ref. [91]. Copyright 2020, Wiley. (**d**) Pyramid-shaped P(VDF-TrFE)-based PENGs. Reproduced with permission from Ref. [92]. Copyright 2020, Wiley. (**e**) 3D stacked triboelectric sensor with micro-cone structures for skin-contact physiological signal perception. Reproduced with permission from Ref. [95]. Copyright 2024, Wiley. (**f**) Textile-based TENGs (T-TENGs) with mm-scale frustum structure. Reproduced with permission from Ref. [73]. Copyright 2020, Springer Nature. (**g**) Structure diagram of a TENG with arch-shaped structure. Reproduced with permission from Ref. [96]. Copyright 2017, Elsevier. (**h**) 3D schematic of a flexible comb triboelectric–electret nanogenerator. Reproduced with permission from Ref. [97]. Copyright 2017, Royal Society of Chemistry.

Several strategies have been developed to improve the piezoelectric performance of PENGs, including material enhancement and structural design. The evolution of materials has progressed from inorganic piezoelectric materials such as piezoelectric ceramics to organic piezoelectric polymers, and has advanced to encompass complex piezoelectric composites [47]. For instance, piezoelectricity can be enhanced from the perspective of composite materials by utilizing a variety of filler materials including organic or inorganic nanomaterials, such as piezoelectric ceramics, metal oxides, carbon-based materials, etc. [84,85,86]. Motivated by this, Yang et al. [87] introduced polydopamine (PDA)-modified barium titanate (BTO) into a poly(vinylidene fluoride) (PVDF) matrix. The casted composite membrane exhibited a remarkable piezoelectric output voltage of 9.3 V under the impact force of 12 N, which showed obvious improvement as compared to the pristine PVDF counterparts (Figure 3a). Badatya et al. [88] fabricated a PVDF–carbon nanotube (CNT) foam-based nanogenerator which generates higher output voltage of ~12 V compared to that of a separate PVDF thin film-based generator (~4 V) at a small compressive pressure of 0.02 kgf.

Structural designs can also improve piezoelectric performance, such as nanofibers [89,90], micro-pillars [91], and pyramid structures [92], etc. Yang et al. [93] prepared a three-dimensional hierarchically interlocked PVDF/ZnO nanofiber-based piezoelectric sensor and the pressing results show that the sensitivity of the sensor has been greatly improved to six times that of pure PVDF nanofibers (Figure 3b). Goni Nayeem et al. [94] utilized a 2.5 μm thick PVDF nanofiber membrane as the sensing layer to fabricate an ultrasensitive sensor which demonstrated a high signal-to-noise ratio and good stability, with sensitivity reaching 10,050.6 mV/Pa in the low frequency range (<500 Hz). Chen et al. [91] proposed a highly sensitive flexible sensor based on a polyvinylidene fluoride-trifluoroethylen [P(VDF-TrFE)]/BTO nano-composite microcolumn array, as shown in Figure 3c. Experimental results show that the piezoelectric performance was improved by 7.3 times compared to pure P(VDF-TrFE) films. Lee et al. [92] demonstrated a pyramid-shaped P(VDF-TrFE) film-based piezoelectric nanogenerator (Figure 3d), which was ultrasensitive in response to mechanical deformation.

TENGs are green energy devices that can harness mechanical motion to generate electricity. In order to improve the output performance of TENGs, materials selection and structural design have been employed. For example, Li et al. [95] employed a 3D stacked triboelectric sensor with micro-cone structures for skin-contact physiological signal perception (Figure 3e). The sensor possessed the capability to generate a voltage of 100 V under constant pressure of 80 N. Zhang et al. [73] developed low-cost triboelectric intelligent socks with a mm-scale frustum structure (Figure 3f). Under the high pressure of 244 kPa, the absolute voltage value of the frustum-patterned sensor was increased three-fold due to the enlarged contact areas and the heightened spacing. Jeon et al. [96] proposed a cost-effective, ambient-based fall detection system based on a TENG with an arch-shaped structure (Figure 3g). Under the pressure induced by hand tapping, the TENG achieved a voltage output of 176 V. Zhu et al. [97] reported a flexible comb triboelectric–electret nanogenerator and the results show that a much larger contact area “finger” sliding generated a voltage output of 45 V (Figure 3h).

### 2.2. Self-Powered Wearable Sensors for Gaits Assessment

Self-powered sensors based on piezoelectric and triboelectric sensing mechanisms have garnered significant attention due to their ability to directly convert mechanical energy into electricity. These sensors can be attached to the wearer’s skin to detect and measure mechanical signals such as strain and pressure [98,99]. By capturing these signals, a more comprehensive understanding of gait patterns can be obtained, enabling the identification of gait abnormalities that may indicate underlying health conditions and facilitating the monitoring of rehabilitation program progress [66,100]. This Section focuses on self-powered wearable flexible sensors, including joint, force, and inertial sensors, and explores their potential applications in human gait analysis.

#### 2.2.1. Joint Sensors 

Joint sensors play a pivotal role in the detection of human movement, enabling the measurement of angular displacement, velocity, and applied torque at the joints [101,102,103]. These sensors provide crucial data that enhance the precision and adaptability of robotic systems. The utilization of joint sensors enables a comprehensive analysis of human movement, thereby facilitating advancements in prosthetic limbs and exoskeletons. By integrating sophisticated joint sensor technologies, researchers can achieve higher levels of accuracy in modeling joint dynamics.

**Figure 4:**
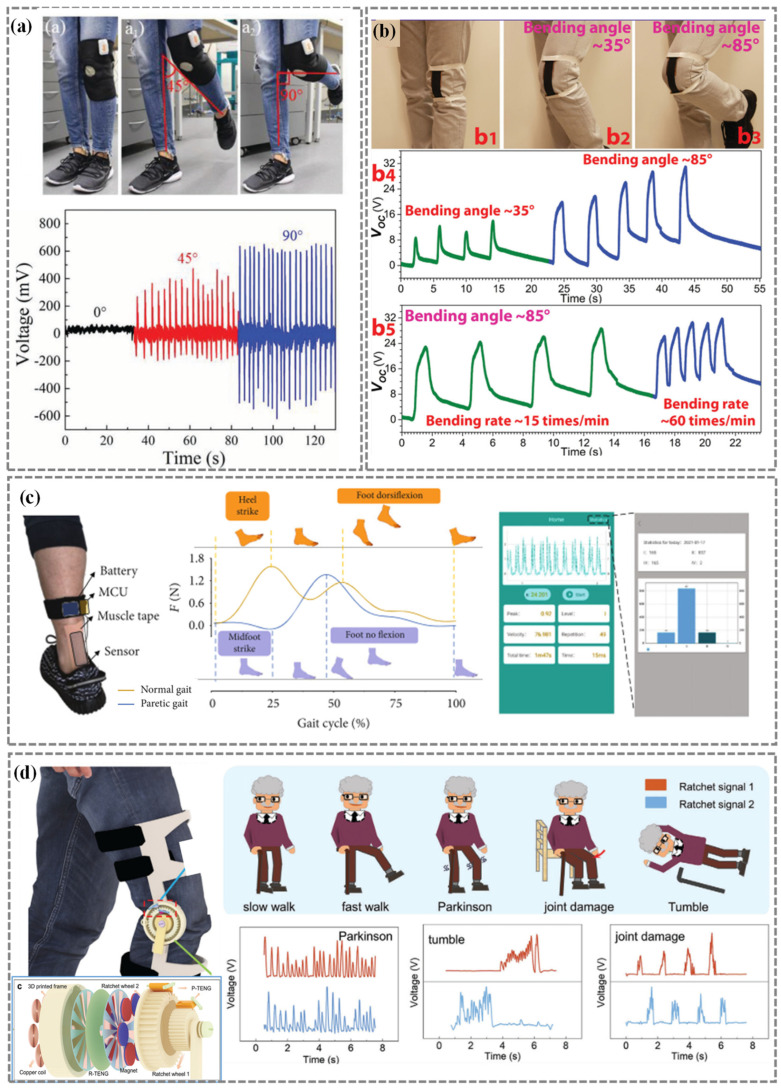
Joint sensors for human motion detection. (**a**) Wearable energy generator based on PVDF/BTO piezoelectric fibers. Reproduced with permission from Ref. [104]. Copyright 2020, Wiley. (**b**) Stretchable-rubber-based triboelectric nanogenerator for body motion detection. Reproduced with permission from Ref. [105]. Copyright 2015, Wiley. (**c**) Comparison of a normal person’s gait with that of a paretic patient detected by the tendon monitoring system. Reproduced with permission from Ref. [68]. Copyright 2021, Science. (**d**) Structure and application scenarios of dual ratchet sensing (DRS) system in detecting states of slow walking, fast walking, onset of Parkinson’s, knee injuries, and falls in elderly individuals by DRS system. Reproduced with permission from Ref. [69]. Copyright 2024, Wiley.

Fatemeh Mokhtari [104] fabricated nanostructured hybrid PVDF/BTO piezoelectric fibers and subsequently knitted the fibers to form a wearable energy generator (Figure 4a). This innovative generator is capable of harnessing energy from knee bending movements during knee bending and unbending to the maximum angle of 0°, 45°, or 90°, which is of great importance for human motion detection. Yi et al. [105] introduced a stretchable rubber-based TENG that not only generates energy but also functions as self-powered multifunctional sensors. The device, comprised of an elastic rubber layer and an aluminum film electrode, has the ability to accurately monitor knee movements at various angles, including 35° and 85°, as well as different bending rates (Figure 4b). In addition to detecting the range of motion of individual joints, it is also important to monitor the movement of multiple joints. Liu et al. [70] developed a self-powered wireless body area network (SWBAN) utilizing contact-separation direct current triboelectric nanogenerators (CSDC-TENGs) for monitoring multi-joint movements in human motion. The SWBAN demonstrated the possibility for tracking joint motion.

Furthermore, the integration of sensors can improve the overall performance and efficiency of the system. Shu et al. [68] introduced a wearable and stretchable bioelectronic patch designed for detecting tendon activities (Figure 4c). The patch is composed of a PVDF piezoelectric material that has been systematically optimized in terms of architecture and mechanics. By integrating the patch with a microcontroller unit (MCU), a tendon real-time monitoring and healthcare system is established, capable of processing collected data and providing feedback for exercise evaluation. Liu et al. [69] presented a self-powered bidirectional knee joint motion monitoring system that utilizes a dual ratchet sensing (DRS) system manufactured by 3D printing technology (Figure 4d). The DRS system is designed to capture the energy generated by knee joint movements and convert it into power for electronic devices without requiring additional metabolic energy from the user. The developed self-powered lower limb status monitoring system is capable of detecting states of slow walking, fast walking, onset of Parkinson’s, knee injuries, and falls in elderly individuals.

#### 2.2.2. Force Sensors

Force-sensitive sensors, typically situated on the sole of footwear, offer invaluable insights into the wearer’s gait pattern by detecting the interaction forces between the foot and the ground [59]. These sensors play a crucial role in capturing detailed biomechanical data, encompassing pressure distribution, ground reaction forces, and temporal parameters of gait. The information gathered from these sensors facilitates a comprehensive analysis of locomotion, which holds paramount importance for diverse applications including clinical gait assessment, rehabilitation, sports performance optimization, and the advancement of adaptive footwear. By converting mechanical stimuli into quantifiable electrical signals, these force-sensitive sensors significantly contribute to our understanding of human movement mechanics, as well as the diagnosis and treatment of gait abnormalities.

**Figure 5:**
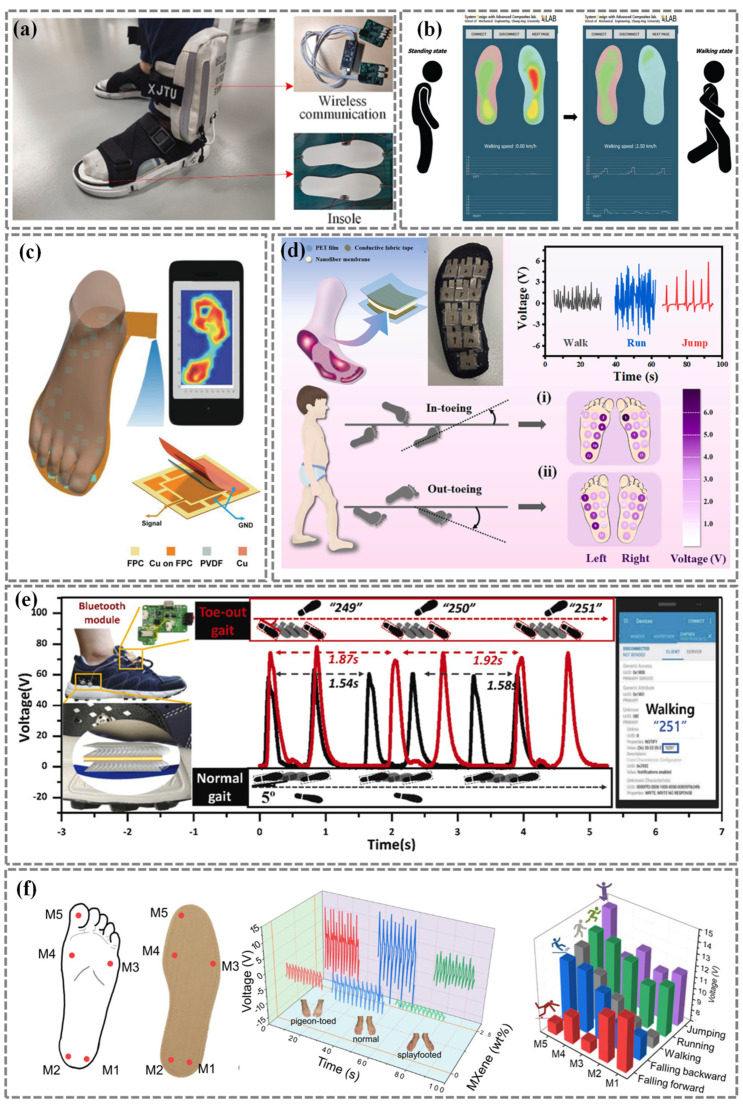
Force sensors for gait recognition based on the piezoelectric principles. (**a**) PVDF strips-based wearable and wireless gait monitoring system. Reproduced with permission from Ref. [106]. Copyright 2022, Elsevier. (**b**) Foot pressure monitoring according to the gait patterns. Reproduced with permission from Ref. [107]. Copyright 2022, Wiley. (**c**) Smart insole based on the fabricated pressure piezo-array for foot pressure distribution monitoring. Reproduced with permission from Ref. [108]. Copyright 2018, Wiley. (**d**) Self-powered sock for walking, running and jumping detection and the gait distribution of the in-toeing and out-toeing walking Reproduced with permission from Ref. [109]. Copyright 2024, Elsevier. (**e**) Application of multi-local strain sensor for gait evaluation. Reproduced with permission from Ref. [110]. Copyright 2020, Elsevier. (**f**) Smart insole integrated with five piezoelectric textile sensors for normal, pigeon-toed, and splayfooted postures detection. Reproduced with permission from Ref. [111]. Copyright 2022, Springer Nature.

Zhao et al. [106] developed a wireless gait monitoring system which consists of a PVDF piezoelectric film sensor, a wireless transmitter, and a receiver. The PVDF piezoelectric film sensor is attached to the sole of the shoe and converts the pressure exerted by the foot into an electrical signal. The proposed system is utilized for normal single walking and cognitive activity (Figure 5a).

In-Jun Jung et al. [107] introduced innovative, self-powered smart shoes with a PVDF ribbon and conductive fabric tape. All gait-related behavioral states, such as foot pressure, walking speed, and energy harvesting, are monitored by the mobile application (Figure 5b). Deng et al. [108] provides an innovative plantar pressure mapping system utilizing a PVDF film sensor array to accurately capture foot pressure distribution and walking motion detection. This sensor array offers a practical solution for gathering biomechanical data during sports/exercise, aiding in injury prevention, and predicting ulceration on the feet (Figure 5c). Meanwhile, Gong et al. [109] presents a PVDF/SiC/FeCl_3_ composite nanofiber-based PENG which exhibited great promise for use in smart socks for gait monitoring, particularly for early corrective measures in preschool children (Figure 5d). Ahn et al. [110] proposed a macro-sized 3D textile structure to improve the piezoelectric performance of PVDF film. The sensor can be used to detect gait pressure and stride length between the normal gait (5°) and toe-out angle gait (25°) using the output voltage (Figure 5e).

Su et al. [111] integrated the electrospun MXene/Sm-PMN-PT/PVDF piezoelectric nanofibers into an insole to form a self-powered gait monitoring system for all-around gait pattern monitoring, walking habit recognition, and Metatarsal bone prediction. Through the dynamic signal mapping between the five units, various gait patterns such as walking, running, jumping, falling forward, and falling backward can be accurately identified and distinguished (Figure 5f).

**Figure 6:**
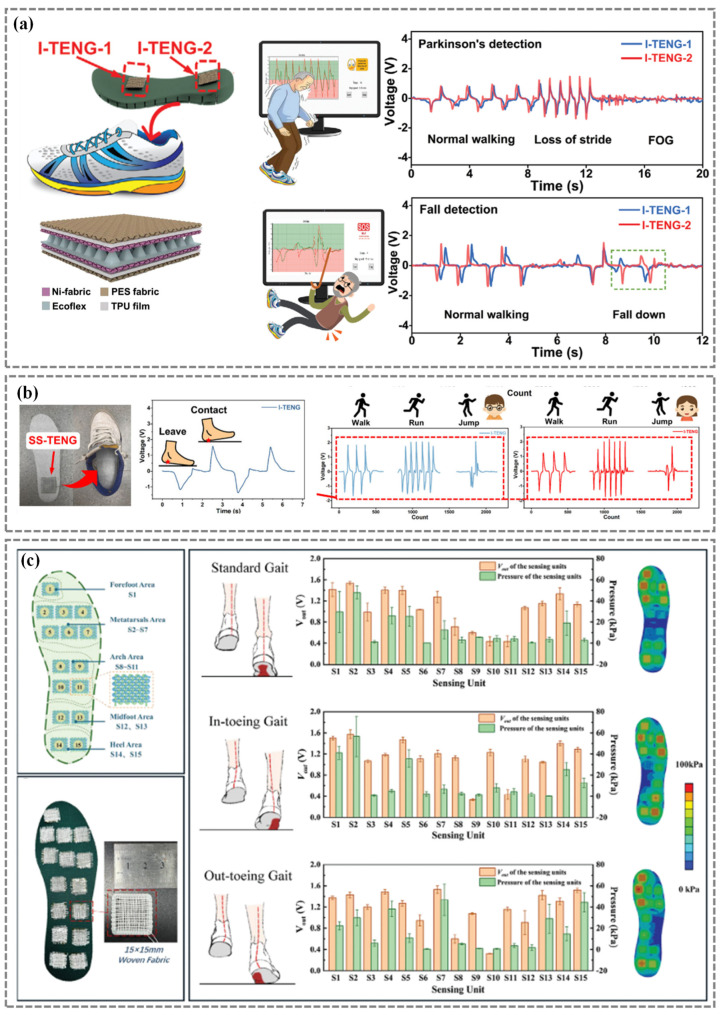
Triboelectric nanogenerators (TENGs) for gait patterns monitoring. (**a**) TENG-based smart insole to detect Parkinson’s symptoms and fall. Reproduced with permission from Ref. [72]. Copyright 2022, Wiley. (**b**) Gait analysis based on TENG smart insole for real-time walking, jogging, and jumping states detection. Reproduced with permission from Ref. [77]. Copyright 2024, Wiley. (**c**) Polyadiohexylenediamine (PA66)/PVDF nanofibers coaxial yarns based TENGs for standard, in-toeing, and out-toeing gait. Reproduced with permission from Ref. [112]. Copyright 2024, Wiley.

TENGs, when installed in the sole or heel of a shoe, have the ability to precisely detect variations in pressure as the feet come into contact with the ground. Zhang et al. [72] introduced wearable TENG-based devices to capture gait motions. The insole equipped with two TENG sensors can be used to detect Parkinson’s symptoms and falls (Figure 6a).

Xu et al. [77] introduced an innovative method for tracking diverse human behaviors by integrating a TENG sensor into an insole. The results show that the sensor can be used for personalized motion monitoring such as walking, jogging, and jumping states of the two participants (Figure 6b).

Wang et al. [112] innovated the use of PVDF and Polyadiohexylenediamine (PA66)-based nanofibers in coaxial yarns to improve the performance of TENGs in capturing human body motion signals. These advanced textiles are adept at real-time monitoring of standard, in-toeing and out-toeing gait (Figure 6c).

#### 2.2.3. Inertial Sensors

The integration of inertial sensors for human motion detection signifies a crucial advancement in the realm of health and activity monitoring [113]. With a focus on precision and efficiency, these sensors facilitate a comprehensive analysis of diverse physical movements, thereby contributing to the development of technologically driven solutions for personalized motion tracking. The inertial sensor includes accelerometer and gyroscope. By harnessing the data acquired from inertial sensors, researchers can effectively capture and interpret intricate details of human motion, thereby paving the way for enhanced techniques in health management and activity monitoring within the domain of Artificial Intelligence and the Internet of Things (AIoT) [114,115,116].

**Figure 7:**
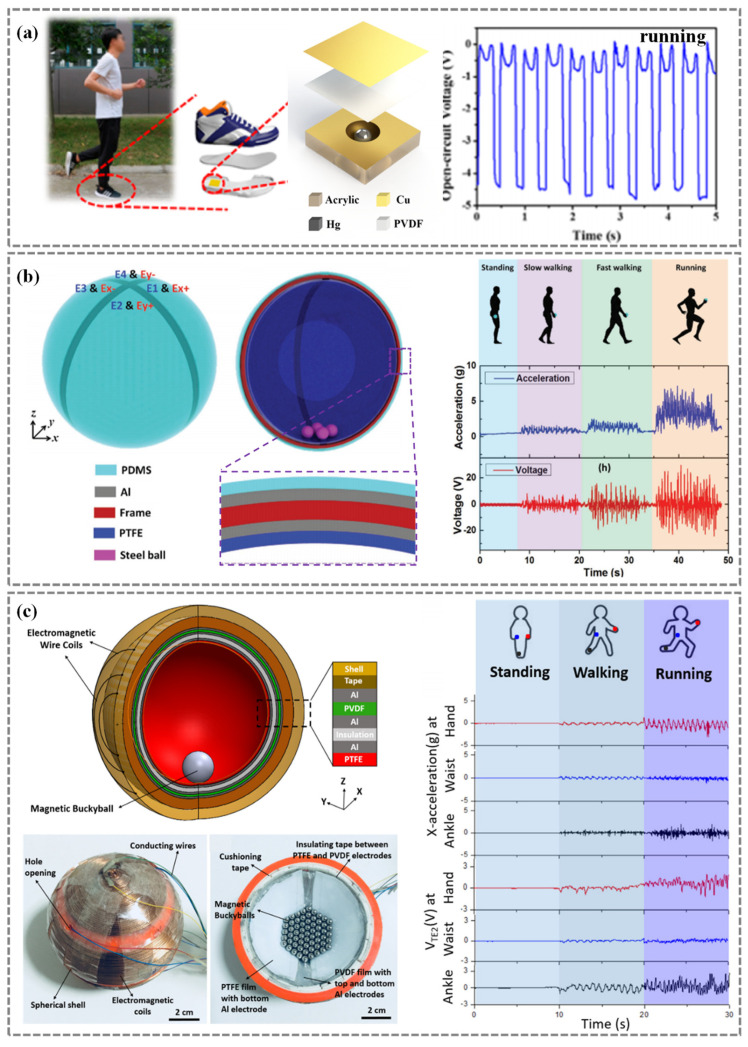
Self-powered inertial sensors for human gait analysis. (**a**) Gait analysis of human running. Reproduced with permission. from Ref. [74]. Copyright 2017, American Chemistry Society. (**b**) Self-powered gyroscope ball for four human activity states detection such as, standing, walking slowly, walking fast, and running. Reproduced with permission from Ref. [76]. Copyright 2017, Wiley. (**c**) Self-powered 3D activity inertial sensor monitors three types of exercise (standing, walking, and running). Reproduced with permission from Ref. [75]. Copyright 2019, Elsevier.

For instance, Zhang et al. [74] presented a self-powered acceleration sensor comprising a liquid metal mercury droplet (LMMD) and a nanofiber-networked PVDF (nn-PVDF) film (Figure 7a). The combination of the ultrahigh surface-to-volume ratio of the nn-PVDF film and the exceptional characteristics of the LMMD resulted in impressive performance metrics, which was successfully employed for real-time measurement of human running. Li et al. [117] developed a novel smart wearable sensor (SWS) utilizing a dual-channel triboelectric nanogenerator integrated into a real-time health monitoring system. The SWS is versatile and can be attached to ankles, shoes, or other body parts and clothing, converting mechanical stimuli into electrical signals. Through the analysis of these signals, the SWS continuously and accurately monitors and differentiates a range of motion states, such as stepping, walking, running, jumping and falling down. 

Furthermore, Shi et al. [76] proposed a 3D symmetric TENG-based gyroscope ball (T-ball) that combined the functionalities of energy harvesting and self-powered sensing for comprehensive motion monitoring, encompassing multi-axis acceleration and rotation. Notably, the T-ball showcased excellent performance in applications foe detection human motion of standing, walking slowly, walking fast, and running (Figure 7b).

Meanwhile, Koh et al. [75] introduced a novel self-powered 3D activity inertial sensor (3DAIS) designed for comprehensive multi-axis acceleration and rotation inertial sensing capabilities. The 3DAIS comprised magnetic bucky balls enclosed within a 3D-printed spherical shell, layered with Polytetrafluoroethylen (PTFE), PVDF, and Al films on the internal surfaces, while wire coils are wound externally. Through successful experimental trials, the device showcased exceptional performance in monitoring human activity states of standing, walking, and running (Figure 7c).

Table 1 provides a summary of representative joint, force, and inertial sensors, with their features based on different sensing mechanisms. As can be seen, in order to maintain performance over prolonged use, these sensors have opted for resilient and flexible materials, such as flexible substrates and high-performance sensing components.

## 3. Human Gait Analysis Algorithms

### 3.1. Gait Monitoring Algorithms

Self-powered sensors have demonstrated significant potential in monitoring human gait information, presenting a noninvasive and convenient approach to gather data on diverse aspects of walking patterns [118,119]. These sensors hold the capability to capture valuable information, including step count, stride length, walking speed, and gait asymmetry, thereby offering valuable insights for medical research, sports performance analysis, and rehabilitation purposes [120,121].

The conventional strategy for gait analysis entails the manual extraction of shallow features, such as frequency, amplitude, peak interval, and holding time, from individual waveforms. However, this approach fails to identify subtle differences among complex features and is highly vulnerable to environmental variations, consequently leading to reduced accuracy in recognition.

The advancement of artificial intelligence has brought forth powerful tools in the form of machine learning for the monitoring of human motion information. Through the utilization of machine learning algorithms, it has become feasible to precisely track and analyze diverse facets of human movement, including gait patterns, posture, and gestures. This technology holds immense potential in enhancing the accuracy and efficiency of human motion monitoring, thereby enabling more personalized and effective healthcare interventions. To date, fast Fourier transform (FFT), calculations and the convolutional neural networks (CNNs) method have been employed for gait monitoring [120,121].

### 3.2. Application of Algorithms in Gait Evaluation 

**Figure 8:**
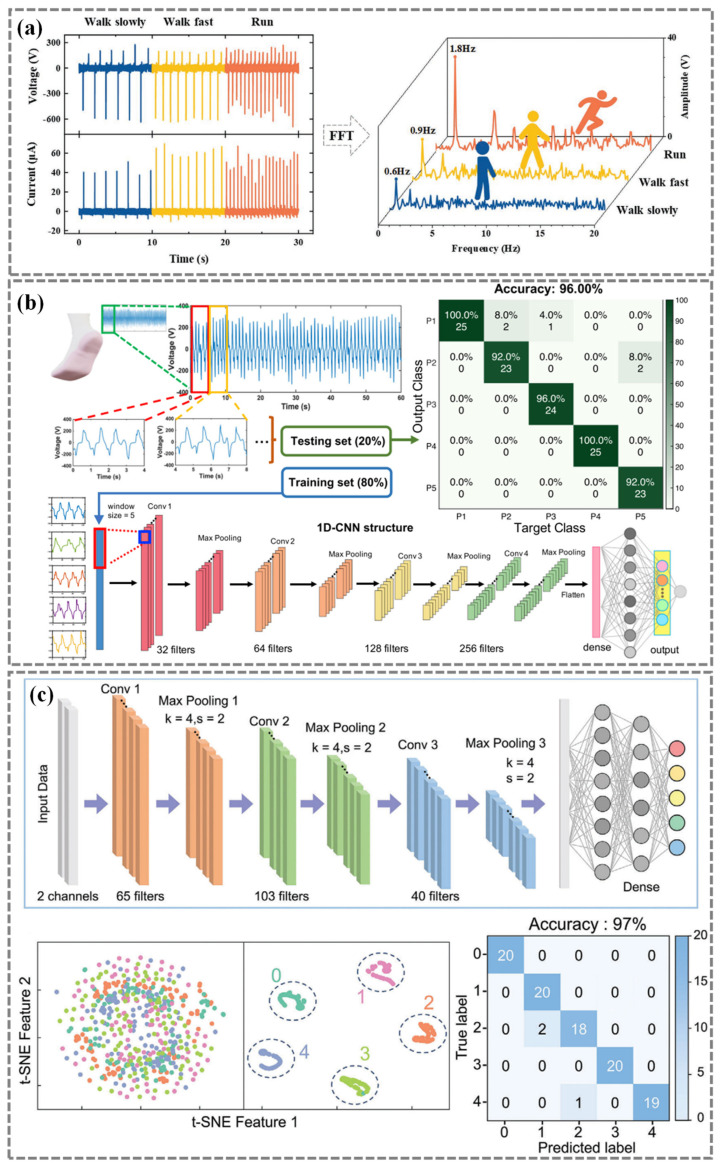
Gait evaluation algorithms. (**a**) Human body movement detection using the fast Fourier transform (FFT) calculation. Reproduced with permission from Ref. [71]. Copyright 2022, Wiley. (**b**) Gait identification using the 1D CNN structure and the confusion map of gait patterns prediction. Reproduced with permission from Ref. [73]. Copyright 2020, Springer Nature. (**c**) Schematics of the application of the CNN algorithm in conjunction with Bayesian optimization and the classification results. Reproduced with permission from Ref. [69]. Copyright 2024, Wiley.

Walking patterns have specific periodicities, with the period being the reciprocal of the frequency. This means that by observing the frequency, the gait state can be inferred. Yang et al. [71] employed the fast Fourier transform (FFT) calculation to obtain the specific frequency (Figure 8a). The experimental results show that the frequencies of walking, brisk walking, and running are 0.6, 0.9, and 1.8 Hz, respectively. 

Meanwhile, Shi et al. [122] presented an intelligent floor monitoring system that integrated self-powered triboelectric floor mats and data analysis based on deep learning. For a dataset consisting of 10 individuals with 1000 data samples, a CNN model was designed to achieve high recognition performance. After training the model for 50 epochs, the average prediction accuracy for recognizing specific walking gaits can reach 96.00%, demonstrating high accuracy in real-time practical scenarios.

Zhang et al. [73] proposed a deep learning-enabled sock which offers an alternative solution for the detection and analysis of the individual gait (Figure 8b). The model achieved an impressive 96% identification accuracy of differentiating gait patterns among five participants and successfully detected five distinct human activities (leap, run, slide, jump, and walk) with 100 training samples for each movement, achieving an accuracy of 96.67%. This innovative approach demonstrated the potential of leveraging deep learning techniques for precise and comprehensive gait analysis, paving the way for improved monitoring and understanding of human movement.

To accurately identify different states of the knee during walking, Liu et al. [69] collected 100 sets of data representing these five knee states including slow walking, fast walking, onset of Parkinson’s disease, knee injury, and falls. From this comprehensive dataset, 10% of the data was extracted as the final test set to evaluate the model’s generalization ability; the remaining 90% of the data was used for model training and validation. Through the amalgamation of convolutional neural network machine learning algorithms with Bayesian optimization techniques, the system can discern up to 97% of knee joint movements (Figure 8c).

## 4. Challenges and Perspectives in Exoskeleton Sensor Technology

Despite the remarkable progress in wearable sensor technology, numerous challenges persist. A key challenge in exoskeleton sensor technology is the integration of wearable sensors into exoskeletons. When exoskeletons and conventional clothes such as shoes are different, these sensors need to be seamlessly integrated into the exoskeleton to capture a diverse array of data, encompassing muscle activity, joint angles, pressure distribution, and balance, which is vital to ensure the efficiency and safety of exoskeleton systems. Besides, achieving this integration necessitates striking a delicate balance between sensor precision and wearability, as bulky or intrusive sensors can hinder natural motion and compromise user comfort. There is a lack of focus on user experience and comfort, especially with wearable technologies like exoskeletons.

Furthermore, the matter of power efficiency presents a substantial hurdle in the advancement of exoskeleton sensor technology. It is imperative to ensure that sensors consume minimal power while delivering real-time feedback, as this prolongs the operational duration of the device and reduces the necessity for frequent recharging or battery replacement. Innovations in energy-efficient sensor technologies, including low-power microelectromechanical systems (MEMS) and wireless communication protocols, hold promising potential in addressing this challenge, thereby establishing the groundwork for more sustainable and practical exoskeleton applications.

Another crucial factor influencing the progress of exoskeleton sensor technology is the realm of data processing and interpretation. Given that sensors generate substantial volumes of intricate biomechanical data, the employment of effective algorithms and signal processing techniques becomes imperative for extracting meaningful information, comprehending user intent, and enabling seamless human-machine interaction. Machine learning approaches, such as neural networks and pattern recognition algorithms, assume a pivotal role in enhancing sensor accuracy, predicting user movements, and optimizing exoskeleton performance in alignment with individual preferences and physiological feedback.

Looking forward, the future of exoskeleton sensor technology in gaits monitoring is set to follow several key directions. Firstly, sensor miniaturization is anticipated to enhance wearability and comfort by creating smaller, lighter, and less obtrusive sensors that seamlessly integrate into everyday objects like clothing. Secondly, ensuring the stability of sensor performance is paramount. This can be bolstered through considerations including material selection, structural design enhancements, effective packaging methods, and seamless circuit integration. Additionally, advancements in 3D gait analysis enabled by depth-sensing technologies and computer vision algorithms promise detailed insights into joint movements and posture dynamics, transforming clinical assessments and rehabilitation practices.

While exoskeleton sensor technology poses a range of challenges regarding sensor precision, power efficiency, and data processing, its transformative potential and expanding horizons highlight a future abundant with opportunities to enhance human performance and well-being. By tackling these challenges head-on and embracing emerging technological trends, the integration of advanced sensors into exoskeletons holds the promise of unlocking novel frontiers in wearable robotics. This integration paves the way for a world where humans and machines collaborate harmoniously, leading to unprecedented achievements.

## 5. Conclusions

Exoskeleton sensors assume a critical role in facilitating the provision of effective and safe assistance to users, thereby underscoring their significance. As sensor technology progresses, exoskeletons are poised to become increasingly capable and user-friendly, thereby unlocking fresh avenues for rehabilitation, assistive technologies, and human augmentation. The integration of advanced sensors into exoskeletons has ushered in novel possibilities for augmenting human capabilities and enhancing overall quality of life. With the ongoing evolution of sensor technology, we anticipate the emergence of even more sophisticated and responsive exoskeletons that will revolutionize diverse industries, including healthcare, manufacturing, and emergency response.

## Figures and Tables

**Table 1:** Summary of the sensors for human gaits detection with their features.

Authors	Materials	Measured Parameter	Sensitivity	Response Time	Cyclic Stability	Features
Mokhtari et al. [104]	PVDF/BTO	Bending angle	10 V N^−1^	2.5 s	1000	High sensitivity
Yi et al. [105]	Stretchable rubber	Bending angle	-	0.0091 s	5000	High elasticity
Shu et al. [68]	PVDF	Bending angle	8.605 V N^−1^	0.018 s	20,000	High resolution
Liu et al. [69]	FEP/Ag	Bending angle	-	-	10,000	High precision
Jung et al. [107]	PVDF	Foot pressure	-	<0.1 s	200 s	High electromechanical performance
Deng et al. [108]	PVDF	Foot pressure	0.008 V kPa^−1^	0.055 s	72,000	High endurance
Gong et al. [109]	PVDF/SiC/FeCl_3_	Foot pressure	-	-	5000	Moisture resistance
Ahn et al. [110]	PVDF	Foot pressure	0.006 V kPa^−1^	-	4000	Two modes detection
Zhang et al. [72]	TPU/PES/Ni/ Ecoflex	Foot pressure	-	<0.91 s	5000	High accuracy
Xu et al. [77]	Ecoflex/Galinstan	Foot pressure	2.47 V N^−1^	<0.5 s	5000	High accuracy
Wang et al. [112]	PVDF/PA66	Foot pressure	8.36 V kPa^−1^	<0.02 s	10,000	High sensitivity
Zhang et al. [74]	PVDF/Acrylic/Cu/ Hg	Acceleration	0.26 V·s/m^2^	-	200,000	High stability
Li et al. [117]	PTFE	Acceleration	-	<0.1 s	1000	High sensitivity
Shi et al. [76]	PTFE/PDMS/Al/ Steel ball	Inertia	3.62 V g^−1^ (z-acceleration)	-	10,000	First TENG-based gyroscope ball
Koh et al. [75]	PTFE/PVDF/Al/ Buckyball	Inertia	0.79 V g^−1^(z-acceleration)	-	-	6-axis inertial detection

Authors

Materials

Measured Parameter

Sensitivity

Response Time

Cyclic Stability

Features
